# Nursing Workload Prediction for Upcoming Shifts: A Retrospective Observational Exploratory Study in the Postoperative and Intensive Care Unit

**DOI:** 10.1155/2024/9703289

**Published:** 2024-01-08

**Authors:** Ann-Margret Hasselgård, Siv K. Stafseth, Øyvind Kirkevold

**Affiliations:** ^1^Postoperative/Intensive Care Unit, Diakonhjemmet Hospital, Oslo, Norway; ^2^Lovisenberg Diaconal University College, Oslo, Norway; ^3^Centre for Patient Centered Heart and Lung Research, Department of Cardiothoracic Surgery, Oslo University Hospital, Oslo, Norway; ^4^The Norwegian National Centre for Ageing and Health, Vestfold Hospital Trust, Tønsberg, Norway; ^5^Research Center for Age-Related Functional Decline and Disease (AFS), Innlandet Hospital Trust, Brumunddal, Norway; ^6^Norwegian University of Science and Technology (NTNU), Department of Health Sciences in Gjøvik, Trondheim, Norway

## Abstract

**Aims:**

This study aimed to explore workload whether Nursing Activities Scores on one shift could predict workload for the next shift.

**Method:**

This was a retrospective observational exploratory study of cross-sectional design carried out in a postoperative and intensive care unit at a local, nonprofit hospital in Norway. Data were collected from the hospital's internal database from January 1st to June 30th, 2016.

**Results:**

A total of 2,695 patients and 5,916 Nursing Activities Scores were included. The model could predict a 55.1% to 66.9% variation in Nursing Activities Scores for the next shift. When the number of patients was added, the model explained up to 80% of the variation.

**Conclusions:**

The Nursing Activities Score can be used to predict nursing workload from one shift to another and as an instrument for managers to adjust their staffing requirements. *Implications for Nursing Management*. Nursing Activities Score assessing nursing workload for all patients in a unit can support the resource planning with accuracy for nurse staffing.

## 1. Background

The mix and size of nursing teams have implications for patient safety, quality of work, nurses' well-being, and the cost of patient care. There is increasing pressure on health expenditure, and several international studies have described resource planning in medical and surgical intensive care units (ICUs)[1–3]. There have also been studies on workload on postoperative and medium care units [4–6]. A review of 26 studies from ICUs found an association between the patient-per-nurse ratio and adverse events such as infections, postoperative complications, unplanned extubations, and burnout syndrome in nurses [7]. A study from 15 Dutch ICUs found that ICU mortality was associated with the nursing workload [8]. Other studies have found that nursing workload is influenced by patient and nurse characteristics [9, 10], and that nursing workload and perceived workload are related [11].

In many postoperative units/ICUs, patient-per-nurse ratios are fixed per shift. However, the association between patient-per-nurse ratios and workload per patient has been found to be weak [8]. Therefore, using workload in one shift to predict workload in the next shift could be a better way for leaders to prioritize resources. Decock et al. [12] investigated whether nurse-level intensity within an intensity group of patients could predict nurse-level intensity on the next shift and on the same shift the next day [12]. The study divided patients into three categories (i.e., levels of lower, median, and high intensity) and could predict 69.7%–74.0% of workload within the categories.

A reliable and validated instrument can help managers prioritize resources to guide resource planning in the short and long term. The Nursing Activities Score (NAS) is a classification instrument used worldwide in medical and surgical ICUs [13]. The instrument was designed to monitor nursing workload per patient per day and shift in order to plan short- and long-term staffing [1, 12, 14]. The NAS consists of 23 different activities that measure direct and indirect ICU patient care, and the instrument has been validated to calculate 81% of actual nursing time. The items were defined by work sampling, and the score represents the calculated percentage of time one nurse spends on each item. A total score of 100% was defined as equal full-time work for one nurse per shift. However, studies have found that one nurse can manage 61–90% of NAS [15] and that certified intensive care nurses manage a higher workload than registered nurses [9]. Several electronical applications are used to find the operational characteristics of the NAS both in the short and long term [16]. Our study can support the idea of an application using NAS as an active tool in planning resources on a shift-to-shift-basis.

Nursing workload can be used as a factor in nurse staffing, and we suggest considering all short stays in a unit in calculating the workload. Monitoring, hygiene procedures, support and care of patients, and administrative and discharge duties are all part of nursing activities for all patients regardless length of stay, and nurse encounters are time consuming in postoperative units/ICUs. The aims of our study were to explore whether the NAS of one shift could predict the NAS of the next shift and to investigate whether the scores were influenced by patient sex, age, ward, type of admission, and the number of patients.

## 2. Methods

### 2.1. Design and Setting

This retrospective, observational, exploratory study was conducted at the postoperative and ICU at a local hospital in the eastern part of Norway. The hospital provides general services within fields such as internal medicine, surgery, and mental health for 145,000 people. The facility is also responsible for treating older people with hip fractures from a larger area. The postoperative unit/ICU is a 19-bed unit that provides invasive and noninvasive respiratory care, invasive hemodynamic monitoring with an arterial catheter, pulse-induced continuous cardiac output, administration of vasoactive and inotropic medications, and continuous dialysis replacement. The unit admits patients from the emergency room, medical and surgical wards, and the operation theater. The postoperative unit/ICU support the outpatient ward when they have 100% bed occupancy and after closing at night. Medical outpatients who receive electrical cardioversion are treated in the postoperative unit/ICU. The nursing staff consists of 50% registered nurses (RNs) with a bachelor's degree and 50% specialized nurses, some with a master's degree. Specialized nurses have a 1.5-year full-time postgraduate education in critical care nursing, nurse anesthetist, or 1-year full-time postgraduate education in cardiac nursing. Anesthesiologists and medical doctors are responsible for medical treatment. During the daytime, a secretary takes care of administrative paperwork and calls, and a technical assistant is responsible for equipment maintenance, storage, and cleaning. The nurse-per-patient ratio varies from 1 : 3 for postoperative patients to 1 : 1 for ICU patients and 2 : 1 for demanding patients. The unit has more nurses during day shifts on weekdays than on other shifts and weekends. Staffing is flexible, and the manager can adjust for the next shift whenever there is a need for more or fewer nurses.

### 2.2. Sample

Data were collected from an internal database in the hospital's administrative system, anonymized, and transferred to a separate file for statistical analysis. The study period was from January 1 to June 30, 2016, and included 2,695 patients and 5,916 scores of NAS over 182 days. The study included all patients admitted to the unit during this period.

### 2.3. Variables

Study variables included patient age, sex, ward (medical or surgical), type of admission (elective or emergency), type of surgery (gastro surgical, orthopedic, or nonsurgical patients), length of stay in the postoperative unit/ICU, patients per day, and shift (Table 1). The median NAS scored per patient for each shift is presented in Table 2.

### 2.4. Procedure Description

The validity and reliability of NAS has been tested in several Norwegian postoperative units and ICUs [5, 15, 17, 18]. From 2014, nurses at our postoperative/ICU scored the Nursing Activities Score on all patients admitted to the hospital. Before its introduction in 2014, nurses had been educated in using the instrument and subsequently undertook an updated course every year. Nurses responsible for the patient care collected the NAS on paper, and the secretary plotted all the data into an internal database. Data were checked for quality by the first author.

The scores of NAS were measured in three 8 hour (h) shifts per 24 h. Night shift began from midnight until 08:00, day shift from 08:00 to 16:00, and evening shift from 16:00 to midnight. For patients with a short length of stay of <8 h, the database was designed to calculate the shift and 24 h scores according to the time the patient stayed in the unit. For example, if a patient had a 50% NAS and stayed only 3 h, then the shift score would be 18.75% NAS, and the 24 h score would be 6.25% NAS for that patient. This aligns with the results of another study [5]. By including length of stay in NAS, a patient with a high workload and short length of stay could get a low NAS score, and a patient with a low workload and a long length of stay get a relatively high NAS.

### 2.5. Statistical Analysis

The data were analyzed using IBM SPSS Statistics version 25.0. Categorical variables were reported as absolute and relative frequencies and analyzed using the Chi-square or Kruskal–Wallis tests as appropriate. Continuous variables were reported as mean and standard deviation (SD) or median as appropriate. Differences between groups were analyzed with Student's *t*-test or Mann–Whitney *U* test. Furthermore, univariable and multivariable linear regression models were used to investigate whether there was an association between the NAS and individual data.

To explore whether the NAS on one shift could predict the NAS on the next shift, the data were aggregated by day. Patient characteristics were also aggregated and included to investigate whether there was an association between the characteristics and the aggregated NAS. In the aggregated material, the NAS and number of patients were summed, and the average ages were determined. The gender, ward, and type of admission were calculated as an average between 0 and 1, indicating the percentages. To explore the extent to which NAS-night shift could predict NAS-day shift, NAS-day shift was set as the dependent variable and NAS-night shift as the independent variable in the regression analysis. To investigate whether NAS-day shift was associated with patient characteristics, the characteristics of day shifts were included in the model. The same method was used to investigate whether the NAS-day shift could predict the NAS-evening shift and to what extent patient characteristics on the evening shift were associated with NAS. In all analyses, a *p* value <0.05 indicate statistical significance.

### 2.6. Ethical Approval

Ethical standards and regulations were followed. In each case, observations were measured and entered in an electronic system, which recorded and displayed results. The need to require informed consent from patients was waived, and data were anonymized prior to transfer to the research team. The study was approved by the local data protection officer of the hospital on the 4^th^ of July 2018 with case number 18/11020. The decision ruled out bioethics committee.

## 3. Results

### 3.1. Patient Characteristics

Demographic and clinical characteristics of participants are presented in Table 1. Most patients were admitted to the emergency department (61.2%) and surgically treated (77.3%). The length of stay varied from 10 min to 23 days, and 62.7% of the surgical and 40.6% of the medical patients stayed in the unit for less than 4 h (not shown). The NAS per shift is described by sex, ward, type of admission, and type of surgery in Table 2. The patients on night shift had the highest NAS with a median of 55.7%. Patients on evening shift had 21.6%, and patients on day shift a median of 19.6% NAS.

### 3.2. Association between NAS Shift Scores and Individual Patient Data

Ward (*β* = −0.33, *p* < 0.001) and type of admission (*β* = 0.33, *p* < 0.001) had the strongest association with NAS-day shift as the dependent variable in the univariable linear regression on individual data in Table 3. The multivariate model explained 17.4% of the variation in the NAS-day shift. This was also observed with the NAS-evening shift as the dependent variables (ward *β* = −0.40, *p* < 0.001 and type of admission *β* = 0.25, *p* < 0.001), and the multivariate model could explain 19.3% of the variation in NAS-evening shift.

### 3.3. Association between NAS Shift Scores and Aggregated Data

**Figure 1:**
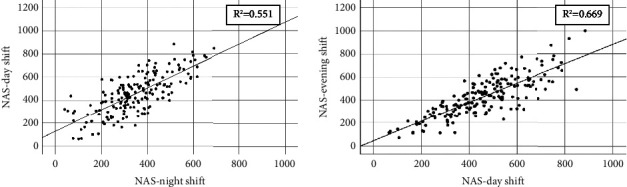
Association between NAS-night shift vs NAS-day shift and NAS-day shift vs NAS-evening shift on aggregated data. NAS, Nursing Activities Score.

Table 4 shows that NAS-nightshift (*β* = 0.74, *p* < 0.001) and the proportion of emergency patients during the day shift (*β* = −0.39, *p* < 0.001) had the strongest association with NAS-day shift as dependent variables on aggregated data in univariable linear regression. The model could explain 73.1% of the variation in NAS-day shift in multivariate linear regression. NAS-night shift and NAS-day shift had a correlation coefficient of 0.74 (not shown), and NAS-night shift could explain 55.1% of the variation in NAS-day shift, Figure 1.

The NAS-day shift had the strongest association with the NAS-evening shift (*β* = 0.82, *p* < 0.001) and the proportion of emergency patients in the evening shift (*β* = −0.32, *p* < 0.001). In multivariate linear regression, the model explained 66.3% of the variation in the NAS-day shift. The NAS-day and evening shifts had a correlation coefficient of 0.82 (not shown), and the NAS-day shift could explain 66.9% of the variation in the NAS-evening shift, Figure 1.

We postulated that NAS shift scores were associated with the number of patients undergoing the shift. With the NAS-day shift as the dependent variable and the NAS-night shift and the number of patients during the day shift as independent variables, multivariate linear regression explained 80.7% of the variation in the NAS-day shift (not shown). If NAS increased by 1% on the night shift, the NAS-day shift increased by 0.8%. For every admitted patient during the day shift, NAS increased by 16.4%. The correlation coefficient between the NAS-day shift and the number of patients on the day shift was 0.70. The same was done with the NAS-evening shift as the dependent variable and the NAS-day shift and number of patients on the evening shift as independent variables. Multivariate linear regression explained 70.4% of the variation in the NAS-evening shift. If NAS increased by 1% on the day shift, the NAS-evening shift would increase by 0.7%. For every patient admitted during the evening shift, the NAS increased by 10.8%. The correlation coefficient between the NAS-evening shift and the number of patients on evening shift was 0.70 (not shown).

## 4. Discussion

This study found a strong association between the NAS in one shift and the NAS in the next shift. When the number of patients in the next shift was added to the model, the NAS-night shift could explain up to 80% of the variation of NAS-day shift, and the NAS-day shift could explain 70% of the variation of NAS-evening shift. This was valid for the unit however not for individual patients. In our study, we analyzed the number of patients in the next shift retrospective. That number would be an unknown factor prospectively, but mean number of patients expected for the shift could be included in the calculation. In this study, patient characteristics had no practical impact on the NAS unlike results found in other studies [2, 10]. Results above 70% show that NAS on one shift has a large effect on NAS on the next shift [19] and that the patient classification system, NAS, can support nurses and managers with the allocation of resources from one shift to the next. The NAS is a dynamic bottom-up instrument that can guide resource planning for a postoperative unit/ICU. Studies have found that bottom-up approach could be a more accurate instrument to guide costing approach [18, 20, 21].

To our knowledge, this is the first study to explore whether total NAS in one shift could predict total NAS in the next shift. To get the full overview of workload in a unit, we ask that all patients regardless of the length of stay should be included since everybody receives nursing care. The study by Decock et al. [12] determined care intensity by NAS cut-offs and found a predictivity of 69.7%–74.0% from one shift to the next. The study included all patients (3,295) admitted on weekdays, unlike our study, which also included patients admitted on weekends. The main difference between the studies was the length of stay, with a short median time of 3.4 h, versus Decock et al.'s 2.8 days. The study by Decock et al. does not describe whether patients with a short length of stay were included or whether patients were adults and/or children, surgical, and/or medical [12]. The study confirmed the findings in our study that NAS can substantially contribute to predicting workload on the next shift.

The staff in charge use their professional judgement to estimate if available resources and expected use of resources agree. Electronic systems are already used to operational NAS trends and characteristics [16]. A mobile NAS application set up to calculate and predict the workload for the next shift could confirm the professional judgement in allocation of resources for the next shift.

The length of stay in our study is in line with a study from a postoperative unit that included all patients with a length of stay of more than 1 h [6]. Other studies have excluded patients with length of stay <4–24 h [8, 15, 22, 23]. We argue that all patients receiving care from a nurse should be included regardless of the length of stay. Not to include all admitted patients could unintentionally exclude a significant amount of workload. Several studies have found an association between the NAS and length of stay [1, 6, 10, 23]. Our study could not analyze this association because the length of stay was included and calculated within the NAS.

In our study, older patients and men were associated with higher scores of NAS when individual data were analyzed. This association was small (not significant), which is in line with other studies [1, 6, 10, 23, 24]. Furthermore, surgical patients had lower scores than medical patients which is in line with the findings of a study by Moghadam et al. [10]. Type of surgery could explain the lower scores for surgical patients as the hospital does not perform heart-, lung-, neuro-, or trauma surgery. There are no gynecological or children service at the hospital. Lima and Rabelo found an association between NAS, length of stay, and magnitude of surgery [6]. Whether the magnitude of surgery or length of stay had the strongest association with NAS was not explored in our study and requires further research.

We found that the NAS from patients on the night shift had the highest median score (55.6%) versus the patients on the day shift (19.6%). On the other hand, the total scores from all patients (aggregated data) were lowest on night shifts and highest on day shifts. Other studies have found low scores on night shifts and the highest scores during the day shifts [1, 10, 22]. In our study, fewer patients at night could explain the low aggregated scores on the night shifts. This means that the unit needed fewer nurses at night, but patients who stayed in the unit at night received more nursing activities.

In our study, the NAS was scored by nurses responsible for patient care. A study by Stuedahl found poor agreement between scores performed by bedside nurses versus managers and physicians [17] and confirmed the initiation by Miranda et al. that the bedside nurse should perform the NAS on every shift [14].

### 4.1. Limitations

This study had some limitations. Only one unit was included, and the retrospective design could only assess associations, not causalities. Including only patients who stayed over to the next shift could change the results and requires further investigation. The study population was a case mix of medical, surgical, elective, and emergency patients, and further research should be performed on the generalizability of our findings. The NAS does not consider the competence level of nurses, and further research should investigate the associations and factors between competence level, workload, patient safety, and quality of care. The NAS does not include emergency preparedness in the scoring, which are important factors for allocating nurses to the next shift. Our unit included length of stay in the NAS, which makes the scores difficult to compare with earlier studies. The COVID-19 pandemic, with an extraordinary workload worldwide, caused a delay in processing results and writing articles.

The strengths of this study include having a complete dataset for all days and shifts during the study period, and that bedside nurse scored NAS on all patients.

### 4.2. Implications and Recommendations for Practice

This study found that the NAS together with number of patients on the next shift could explain up to 80% of the variation of workload on the next shift. Implications for practice could be that the NAS tool can confirm professional judgement to allocate resources for the upcoming shift. We recommend the development of a mobile NAS application that merge the NAS for one shift, the number of patients expected on the next shift, available nurse staff, and emergency preparedness. This objective application together with professional judgement can empower managers prioritize scarce resources on a shift-to-shift-basis.

## 5. Conclusions

This study found that the NAS can be used to predict nursing workload from one shift to the next and be a beneficial instrument for managers to adjust their staffing requirements. However, patient safety and the best quality of care are also influenced by other factors, such as nurses' qualifications, cooperation among healthcare professionals, and external variables. However, these were not included in the aim of this study and require further research.

## Figures and Tables

**Table 1:** Sociodemographic description of number of patients, age, sex, type of admission, type of surgery, length of stay in hours, number of days, and number of patients per day and shift.

			Medical patients	Surgical patients	*p*
Patients, number (%)		2695	613 (22.7)	2082 (77.3)	

Age, mean (SD^†^)		63.2 (18.9)	59.8 (20.4)	64.2 (18.3)	<0.001^§^

*Sex, number (%)*					
	Male	1257 (46.6)	388 (30.9)	869 (69.1)	
	Female	1438 (53.4)	225 (15.6)	1213 (84.4)	<0.001^¶^

*Type of admission, number (%)*					
	Elective	1047 (38.8)	74 (7.1)	973 (92.9)	
	Emergency	1648 (61.2)	539 (32.7)	1109 (67.2)	<0.001^¶^

*Type of surgery, number (%)*					
	Gastrosurgical			635 (30.5)	
	Orthopedic			1300 (62.4)	
	Nonoperated			147 (7.1)	

Length of stay hours PO/ICU^‡‡^ median (Q1–Q3^‡^)		3.4 (2.1–6.3)	7.2 (2.0–19.8)	3.2 (2.1–5.0)	<0.001^††^

Number of days		182			

*Patients per day and shift, mean (SD* ^*†*^)					
	Total	20.2 (6.7)			
	Night shift	6.6 (2.3)			
	Day shift	13.9 (5.4)			
	Evening shift	12.0 (4.2)			

^†^ = SD-standard deviation, ^‡^ = Q1–Q3-interpercentile range, ^§^ = Student's *T*-test, ^¶^ = Chi-square, ^††^ = Mann–Whitney *U*, ^‡‡^ = PO/ICU-postoperative/intensive care unit. *p* value <0.05 indicates statistical significance.

**Table 2:** Nursing activities scores per patient per shift (night, day, and evening) described by sex, ward, type of admission, and type of surgery.

Categories and variables	NAS^†^-night shift			NAS^†^-day shift			NAS^†^-evening shift		
	*n* ^‡^ (%)	Median (Q1–Q3^§^)	*p*	*n* ^‡^ (%)	Median (Q1–Q3^§^)	*p*	*n* ^‡^ (%)	Median (Q1–Q3^§^)	*p*
NAS^†^ per patient per shift	1204	55.7 (26.1–73.1)		2521	19.6 (8.7–49.1)		2191	21.6 (9.6–58.3)	

*Sex*									
Male	673 (55.9)	58.1 (30.9–78.0)		1276 (50.6)	22.7 (8.8–61.8)		1074 (49.0)	27.1 (10.7–68.8)	
Female	531 (44.1)	51.4 (20.8–68.6)	<0.001^¶^	1245 (49.4)	17.9 (8.6–37.2)	<0.001^¶^	1117 (51.0)	18.6 (8.9–44.7)	<0.001^¶^

*Ward*									
Medical	606 (50.3)	60.1 (41.6–80.7)		859 (34.1)	40.7 (15.1–74.2)		610 (27.8)	59.3 (23.3–86.2)	
Surgical	598 (49.7)	46.3 (14.6–65.6)	<0.001^¶^	1662 (65.9)	15.7 (7.3–30.6)	<0.001^¶^	1581 (72.2)	16.6 (8.4–36.3)	<0.001^¶^

*Type of admission*									
Emergency	1123 (93.3)	55.5 (26.2–74.1)		1535 (60.9)	28.9 (9.9–68.3)		1561 (71.2)	27.5 (10.9–67.9)	
Elective	81 (6.7)	56.0 (10.6–67.1)	0.313^¶^	986 (39.1)	14.3 (7.5–25.1)	<0.001^¶^	630 (28.8)	14.6 (7.0–30.0)	<0.001^¶^

*Surgical*									
Gastrosurgical	263 (43.9)	58.2 (19.4–70.7)		564 (33.9)	14.9 (7.2–48.3)		540 (34.2)	17.7 (8.4–57.7)	
Orthopedic	229 (38.3)	25.1 (9.6–51.9)		940 (56.6)	15.3 (7.4–24.8)		903 (57.1)	15.3 (8.6–27.4)	
Nonoperated	106 (17.8)	56.2 (36.6–83.9)	<0.001^††^	158 (9.5)	31.8 (7.2–69.6)	<0.001^††^	138 (8.7)	42.3 (8.2–81.3)	<0.001^††^

^†^ = NAS-nursing activities score, ^‡^ = *n*-number of NAS per shift, ^§^ = Q1–Q3-interpercentile range, ^¶^ = Mann–Whitney *U*, ^††^ = Kruskal–Wallis test. *p* value <0.05 indicates statistical significance.

**Table 3:** Linear regression by individual data with NAS-day shift and NAS-night shift as dependent variables.

NAS-day shift as a dependent variable	Univariable linear regression				Multivariable linear regression			
	*B* (95% CI)	*β*	*p*	*r* ^2^	*B* (95% CI)	*β*	*p*	Adjusted *R*^2^ = 0.174
Age, day	0.12 (0.05, 0.19)	0.07	<0.001	0.005	0.17 (0.11, 0.23)	0.10	<0.001	
Sex, day (men 0, women 1)	−8.76 (−11.30, −6.23)	−0.13	<0.001	0.018	−5.34 (−7.73, −2.96)	−0.08	<0.001	
Ward, day (medical 0, surgical 1)	−23.39 (−26.03, −20.74)	−0.33	<0.001	0.107	−17.13 (−19.87, −14.38)	−0.24	<0.001	
Type of admission, day (elective 0, emergency 1)	23.34 (20.71, 25.96)	0.33	<0.001	0.108	17.16 (14.48, 19.84)	0.24	<0.001	

NAS-evening shift as a dependent variable	Univariable linear regression				Multivariable linear regression			
	*B* (95% CI)	*β*	*p*	*r* ^2^	*B* (95% CI)	*β*	*p*	Adjusted *R*^2^ = 0.193

Age, day	0.14 (0.07, 0.22)	0.08	<0.001	0.007	0.22 (0.15, 0.28)	0.12	<0.001	
Sex, day (men 0, women 1)	−10.92 (−13.65, −8.19)	−0.17	<0.001	0.027	−6.97 (−9.52, −4.42)	−0.11	<0.001	
Ward, day (medical 0, surgical 1)	−28.65 (−31.43, −25.87)	−0.40	<0.001	0.157	−25.06 (−27.99, −22.12)	−0.35	<0.001	
Type of admission, day (elective 0, emergency 1)	17.61 (14.70, 20.53)	0.25	<0.001	0.060	8.66 (5.79, 11.52)	0.12	<0.001	

NAS, nursing activities score; *B*, unstandardized regression coefficient; CI, confidence interval; *β*, standardized regression coefficient. *p* value <0.05 indicates statistical significance.

**Table 4:** Linear regression on aggregated data with NAS-day shift and NAS-evening shift as dependent variables and NAS-night shift, NAS-day shift, and demographic characteristics as independent variables.

NAS-day shift as dependent variable	Univariable linear regression				Multivariable linear regression			
	B (95% CI)	*β*	*p*	*r* ^2^	B (95% CI)	*β*	*p*	Adjusted *R*^2^ = 0.731
NAS-night shift	0.94 (0.82, 1.07)	0.74	<0.001	0.551	0.97 (0.87, 1.06)	0.76	<0.001	
Age mean, day	−1.11 (−5.59, 3.37)	−0.04	0.625	0.001	1.11 (−1.26, 3.48)	0.04	0.357	
Proportion of women, day	−0.60 (−2.24, 1.04)	−0.05	0.471	0.003	−0.28 (−1.18, 0.63)	−0.03	0.548	
Proportion of surgical patients, day	1.52 (0.14, 2.91)	0.16	0.031	0.026	−0.41 (−1.41, 0.59)	−0.04	0.424	
Proportion of emergency patients, day	−2.81 (−3.79, −1.82)	−0.39	<0.001	0.150	−3.40 (−4.15, −2.65)	−0.47	<0.001	

NAS-evening shift as dependent variable	Univariable linear regression				Multivariable linear regression			
	B (95% CI)	*β*	*p*	*r* ^2^	B (95% CI)	*β*	*p*	Adjusted *R*^2^ = 0.663

NAS-day shift	0.84 (0.75, 0.92)	0.82	<0.001	0.669	0.84 (0.74, 0.94)	0.82	<0.001	
Age mean, evening	0.61 (−3.07, 4.29)	0.02	0.744	0.001	0.88 (−1.32, 3.08)	0.04	0.429	
Proportion of women, evening	−1.03 (−2.47, 0.42)	−0.10	0.164	0.011	−0.34 (−1.23, 0.55)	−0.04	0.448	
Proportion of surgical patients, evening	0.57 (−0.94, 2.08)	0.06	0.457	0.003	0.04 (−1.05, 1.12)	0.003	0.949	
Proportion of emergency patients, evening	−2.97 (−4.26, −1.68)	−0.32	<0.001	0.103	0.10 (−0.94, 1.13)	0.01	0.855	

NAS, Nursing Activities Score; B, unstandardized regression coefficient; CI, confidence interval; *β*, standardized regression coefficient. *p* value <0.05 indicates statistical significance.

## Data Availability

Data were collected from an internal database in the hospital's administrative system.
